# Supplementary material for: Effect of dietary branched chain amino acids on liver related mortality: Results from a large cohort of North American patients with advanced HCV infection
Source: PLoS One. 2023 Apr 25;18(4):e0284739. doi: 10.1371/journal.pone.0284739 (PMC10128927; doi:10.1371/journal.pone.0284739)
Supplement: S1 Table — (DOCX) [file pone.0284739.s001.docx]

**S1 Table. Rates of liver related decompensations according to quartiles of BCAA intake derived from average daily BCAA intake (measured in grams of BCAA per 1000 kcal of daily energy intake).**

| Quartiles and range (g/1000 kcal of energy) of BCAA intake | Variceal bleeding  (N, %) | Ascites  (N, %) | Spontaneous peritonitis  (N, %) |
| --- | --- | --- | --- |
| 1  (3.0-5.6) | 5  (3.0%) | 18  (11%) | 1  (0.6%) |
| 2  (5.6-6.4) | 1  (0.6%) | 11  (6.7%) | 0 |
| 3  (6.4-7.4) | 4  (2.4%) | 16  (9.8%) | 2 (1.2%) |
| 4  (7.4-34.8) | 6  (3.7%) | 10  (6.1%) | 1 (0.6%) |
