# Supplementary material for: Effect of dietary branched chain amino acids on liver related mortality: Results from a large cohort of North American patients with advanced HCV infection
Source: PLoS One. 2023 Apr 25;18(4):e0284739. doi: 10.1371/journal.pone.0284739 (PMC10128927; doi:10.1371/journal.pone.0284739)
Supplement: S2 Table — (DOCX) [file pone.0284739.s002.docx]

**S2 Table. Crude and adjusted hazard ratios of liver-related death or transplantation according to quartiles of BCAA intake derived from average daily energy adjusted BCAA intake (measured in grams of BCAA per 1000 kcal of daily energy intake).**

|  | Crude HR  (95% CI) | ^a^AHR  (95% CI) |
| --- | --- | --- |
| BCAA intake categories |  |  |
| 1 | 1 | 1 |
| 2 | 1.04 (0.60-1.81) | 0.92 (0.49-1.75) |
| 3 | 1.03 (0.59-1.80) | 0.97 (0.50-1.90) |
| 4 | 0.97 (0.55-1.70) | 1.03 (0.52-2.05) |
| Age | 1.01 (0.98-1.04) | 1.01 (0.97-1.04) |
| Sex |  |  |
| Male | 1 | 1 |
| Female | 0.85 (0.54-1.34) | 0.82 (0.46-1.47) |
| Race |  |  |
| White | 1 | 1 |
| Black | 1.30 (0.78-2.17) | 0.97 (0.51-1.85) |
| Hispanic | 1.01 (0.44-2.33) | 0.76 (0.29-1.97) |
| Other | 1.86 (0.67-5.10) | 1.97 (0.59-6.60) |
| Cirrhosis | 3.04 (2.00-4.63)^b^ | 2.96 (1.84-4.74)^b^ |
| Diabetes | 0.84 (0.49-1.44) | 0.91 (0.47-1.75) |
| Lifetime number of alcohol drinks | 1.00 (1.00-1.00) | 1.00 (0.94-1.04) |
| Body mass index | 0.99 (0.95-1.03) | 0.99 (0.94-1.04) |
| Smoking | 1.50 (1.01-2.22)**^b^** | 1.52 (0.94-2.48) |
| Self-rated health status |  |  |
| Excellent | 1 | 1 |
| Very good | 3.32 (0.44-24.7) | 3.80 (0.49-29.6) |
| Good | 4.11 (0.57-29.8) | 4.08 (0.53-31.1) |
| Fair | 3.89 (0.52-28.8) | 3.94 (0.50-31.1) |
| Poor | 5.48 (0.64-47.0) | 7.34 (0.78-69.3) |
| Peg-Interferon treatment | 1.01 (0.68-1.50) | 0.97 (0.62-1.51) |
| Duration of HCV infection | 1.01 (0.99-1.04) | 1.01 (0.98-1.04) |
| Coffee intake |  |  |
| Nondrinker | 1 | 1 |
| < 1 cup/day | 1.70 (0.90-3.21) | 1.73 (0.85-3.51) |
| 1-2 cups/day | 1.06 (0.56-2.00) | 1.01 (0.49-2.09) |
| ≥ 3 cups/day | 0.86 (0.34-2.15) | 0.78 (0.29-2.14) |
| Calorie intake | 1.00 (1.00-1.00) | 1.00 (1.00-1.00) |
| Cholesterol intake | 1.00 (1.00-1.00) | 1.00 (1.00-1.00) |

^a^Full model adjusted for age, sex, race, BMI, diabetes, lifetime alcohol intake, smoking status, coffee intake, self-reported health status, cirrhosis status, duration of infection, peginterferon treatment group, daily average energy intake and daily average cholesterol intake.

^b^ Indicates statistical significance at P<0.05
