# Supplementary material for: Effect of dietary branched chain amino acids on liver related mortality: Results from a large cohort of North American patients with advanced HCV infection
Source: PLoS One. 2023 Apr 25;18(4):e0284739. doi: 10.1371/journal.pone.0284739 (PMC10128927; doi:10.1371/journal.pone.0284739)
Supplement: S3 Table — (DOCX) [file pone.0284739.s003.docx]

**S3 Table. Crude and adjusted hazard ratios of first liver related decompensations (including first event of variceal bleeding, ascites, spontaneous peritonitis or encephalopathy) according to quartiles of BCAA intake derived from average daily BCAA intake (measured in grams of BCAA per 1000 kcal of daily energy intake).**

|  | Crude HR  (95% CI) | ^a^AHR  (95% CI) |
| --- | --- | --- |
| BCAA intake categories |  |  |
| 1 | 1 | 1 |
| 2 | 0.70 (0.35-1.38) | 0.63 (0.30-1.36) |
| 3 | 1.10 (0.60-2.03) | 1.05 (0.51-2.16) |
| 4 | 0.97 (0.52-1.82) | 0.91 (0.42-1.95) |
| Age | 0.99 (0.96-1.03) | 0.99 (0.95-1.03) |
| Sex |  |  |
| Male | 1 | 1 |
| Female | 1.27 (0.78-2.05) | 1.07 (0.58-1.96) |
| Race |  |  |
| White | 1 | 1 |
| Black | 1.31 (0.72-2.38) | 1.18 (0.58-2.43) |
| Hispanic | 2.08 (1.02-4.22)^b^ | 1.04 (0.44-2.45) |
| Other | 0.60 (0.08-4.37) | 0.83 (0.11-6.25) |
| Cirrhosis | 3.26 (2.02-5.29)^b^ | 4.27 (2.44-7.48)^b^ |
| Diabetes | 0.95 (0.50-1.81) | 1.09 (0.52-2.28) |
| Lifetime number of alcohol drinks | 1.00 (1.00-1.00) | 1.00 (1.00-1.00) |
| Body mass index | 1.05 (1.01-1.09) | 1.03 (0.99-1.08) |
| Smoking | 0.88 (0.52-1.48) | 0.82 (0.44-1.51) |
| Self-rated health status | 1.57 (1.21-2.04)^b^ | 1.44 (1.06-1.96)^b^ |
| Peginterferon group | 1.31 (0.83-2.07) | 1.06 (0.64-1.77) |
| Duration of HCV infection | 0.99 (0.96-1.02) | 0.99 (0.96-1.02) |
| Coffee intake |  |  |
| Nondrinker | 1 | 1 |
| < 1 cup/day | 0.67 (0.34-1.28) | 0.60 (0.30-1.23) |
| 1-2 cups/day | 0.57 (0.31-1.05) | 0.54 (0.27-1.09) |
| ≥ 3 cups/day | 0.43 (0.16-1.17) | 0.42 (0.14-1.28) |
| Calorie intake | 1.00 (1.00-1.00) | 1.00 (1.00-1.00) |
| Cholesterol intake | 1.00 (1.00-1.00) | 1.00 (1.00-1.00) |

^a^Full model adjusted for age, sex, race, BMI, diabetes, lifetime alcohol intake, smoking status, coffee intake, self-reported health status, cirrhosis status, duration of infection, peginterferon treatment group, daily average energy intake and daily average cholesterol intake.

^b^ Indicates statistical significance at P<0.05
