# Supplementary material for: Effect of dietary branched chain amino acids on liver related mortality: Results from a large cohort of North American patients with advanced HCV infection
Source: PLoS One. 2023 Apr 25;18(4):e0284739. doi: 10.1371/journal.pone.0284739 (PMC10128927; doi:10.1371/journal.pone.0284739)
Supplement: S4 Table — (DOCX) [file pone.0284739.s004.docx]

**S4 Table. Risk of liver-related death or transplantation according to quartiles of average BCAA/total protein/caloric intake (measured in grams BCAA per grams total protein per 1000 kcal)**

| Quartiles of daily BCAA/total protein/caloric intake | HR  (95% CI) | ^a^AHR  (95% CI) | ^a^P-value and AHR for trend |
| --- | --- | --- | --- |
|  |  |  |  |
| 1 | 1.00 | 1.00 |  |
| 2 | 1.16  (0.66-2.06) | 1.18  (0.50-2.78) |  |
| 3 | 1.61  (0.94-2.76) | 1.66  (0.61-4.54) |  |
| 4 | 0.88  (0.48-1.61) | 1.16  (0.33-4.07) | P=0.80,  1.05  (0.72-1.52) |

^a^Adjusted for age, sex, race, BMI, diabetes, lifetime alcohol intake, smoking status, coffee intake, self-reported health status, cirrhosis status, duration of infection, peginterferon treatment group, daily average energy intake and daily average cholesterol intake. Only subjects with complete data (N=585) were included in the multiple Cox regression model.
