# Supplementary material for: Effect of dietary branched chain amino acids on liver related mortality: Results from a large cohort of North American patients with advanced HCV infection
Source: PLoS One. 2023 Apr 25;18(4):e0284739. doi: 10.1371/journal.pone.0284739 (PMC10128927; doi:10.1371/journal.pone.0284739)
Supplement: S5 Table — (DOCX) [file pone.0284739.s005.docx]

**S5 Table. Risk of liver-related death or transplantation according to quartiles of average daily absolute BCAA intake without accounting for total energy intake (measured in grams of BCAA)**

| Quartiles of absolute daily BCAA intake | HR  (95% CI) | ^a^AHR  (95% CI) | ^a^P-value and AHR for trend |
| --- | --- | --- | --- |
|  |  |  |  |
| 1 | 1.00 | 1.00 |  |
| 2 | 1.32  (0.75-2.34) | 0.90  (0.45-1.78) |  |
| 3 | 1.27  (0.72-2.24) | 0.97  (0.47-2.01) |  |
| 4 | 1.32  (0.75-2.33) | 1.14  (0.45-2.88) | P=0.77,  1.04  (0.78-1.41) |

^a^Adjusted for age, sex, race, BMI, diabetes, lifetime alcohol intake, smoking status, coffee intake, self-reported health status, cirrhosis status, duration of infection, peginterferon treatment group, and daily average cholesterol intake. Only subjects with complete data (N=585) were included in the multiple Cox regression model.
