# Supplementary material for: Effect of dietary branched chain amino acids on liver related mortality: Results from a large cohort of North American patients with advanced HCV infection
Source: PLoS One. 2023 Apr 25;18(4):e0284739. doi: 10.1371/journal.pone.0284739 (PMC10128927; doi:10.1371/journal.pone.0284739)
Supplement: Attachment — Submitted filename: Response to Reviewers.docx [file pone.0284739.s006.docx]

Review Comments to the Author

Please use the space provided to explain your answers to the questions above. You may also include additional comments for the author, including concerns about dual publication, research ethics, or publication ethics. (Please upload your review as an attachment if it exceeds 20,000 characters)

**Reviewer #1**: In this retrospective cohort study conducted by using the extended follow-up data from the HALT-C trial, the authors suggested that dietary BCAA intake was not associated with liver-related death or liver transplantation. Besides, incidence of HCC or hepatic encephalopathy was not associated with dietary BCAA level.

Major comments
The authors used the extended data from the HALT-C trial, and the patients enrolled in this trial were with hepatic fibrosis (Ishak score 3-4) or compensated cirrhosis (CTP score < 7). According to the previous studies, the effects on BCAAs, which act as pharmaco-nutrients but not medication, on hepatic outcomes were more suggested in patients who had developed cirrhotic complications. The less severity of liver disease in these patients may contribute to the negative findings in this study.

1. The dietary of BCAA was assessed by the food frequency questionnaire, the cornerstone of this study. The authors have to explain how to deal with the potential response bias and recall bias that would have the major influence in this study.

**Response**: We agree with the reviewer that food frequency questionnaire (FFQ) has inherent limitations which may lead to measurement error in the exposure of interest, in this case, dietary BCAA intake. Extensive reviews are available discussing the various ways to account or adjust for measurement error likely result from recall or response bias. Such adjustment always requires a sub-sample of the study population to undergo a more “rigorous” diet assessment, such as multiple-day food diary with interviews (Bennett et al. BMC Medical Research Methodology (2017) 17:146 DOI 10.1186/s12874-017-0421-6). The HALT-C trial, unfortunately, did not obtain such data.

We would like to stress that FFQ is a highly valued tool in assessing the relationship between “usual” diet and disease outcomes. Not only can FFQ be administered in a large study population (in contrast to the more accurate, but cumbersome tools such as food diary), it is particularly valuable when it is administered multiple times, such as in the current study. Repeat measurements can more accurately capture one’s average diet intake over time and may reduce inherent recall bias when only one FFQ was performed. Averaging the intakes from multiple measurements is a well-accepted practice in nutritional epidemiology (Ref 26: Hu FB, et al. Dietary fat and coronary heart disease: a comparison of approaches for adjusting for total energy intake and modeling repeated dietary measurements. Am J Epidemiol 1999;149:531-40).

We have modified our discussion section to reflect the reviewer’s concern.

2. As mentioned in your method, the diet of patients may change overtime, and multiple assessments were applied to calculate the dietary components. However, the detailed information, such as the interval of questionnaire assessment, was not described at all. Considering the long-term follow-up period in your study, the authors have to provide more information about the questionnaire assessment and describe how to ensure the stability of the patients’ dietary habit/ component during the assessment interval. The authors also have to tell how to manage the data if a remarkable change of dietary habit/component was observed in a patient during the long-term follow-up.

**Response**: We agree with the reviewer that more detailed data on BCAA estimates, especially when two FFQ’s are available, should be included for clarification. Firstly, in terms of interval between the two FFQ’s, the second FFQ was completed 661±269 days after the first one, or approximately 22 months. Secondly, among 656 patients in the final study cohort, the median intake of BCAA/1000 kcal estimated from the second FFQ (6.4 g, IQR 5.4 – 7.5 g) was essentially the same compared to the first FFQ (6.4 g, IQR 5.4 – 7.3 g), suggesting minimal change in BCAA intake during the period of two FFQ assessments. Based on detailed description of the HALT-C trial (Ref 23: Lee et al. Control Clin Trials 2004;25:472-92), there was no mention of a specific protocol to ensure stable diet during the study period. Because diet is not the primary exposure of interest of the trial, we suspect no effort was made to counsel patients about dietary behaviors.

We have included the above data in the Methods and Results section.

3. The BCAA intake is positively correlated with total energy intake, which is closely associated with the whole nutritional status and would have substantial impacts on the clinical outcomes. Besides, the BCAA intake amount was also found significantly associated with the dietary amount of aromatic AA that would also affect the presence of HE and other complications. To reduce the overfitting and multicollinearity of these factors, a regularized regression analysis, such as Ridge regression or Lass regression, is more appropriate to investigate the risk of outcomes in this study.

**Response**: We agree with the reviewer that collinearity between variables are important considerations in the analysis of study outcomes. This is particularly relevant in nutritional epidemiology studies where intake of most nutrients are correlated with intake of “total calories” (the more one eats of everything, the more one consumes a particularly nutrient), although to varying degrees depending on the nutrient. One commonly accepted method to address this issue is the nutrient density model employed in the current study. Instead of assessing the effect of absolute intake of BCAA, the exposure variable is “categorized intake of BCAA per 1000 Kcal” where the “collinear” effect of total caloric intake on BCAA consumption is accounted for mathematically (Ref 27: Willett WC, Howe GR, Kushi LH. Adjustment for total energy intake in epidemiologic studies. Am J Clin Nutr 1997;65:1220S-1228S; discussion 1229S-1231S).

In terms of the correlation between calorie intake and “nutritional status” of study subjects, the HALT-C data unfortunately did not assess nutritional status with detailed anthropometric assessment other than BMI, which was not correlated with absolute BCAA intake (Rho=0.004, P=0.9) or BCAA/1000 Kcal (Rho=0.01, P=0.7). Because no patients had hepatic decompensations at the time of study enrollment (only patients with advanced fibrosis or compensated cirrhosis were included), we do not anticipate significant abnormality in the overall nutritional status of study subjects. We, however, do agree with the reviewer that this is potentially an important issue and should be assessed carefully in future studies.

With regard to the utility of regularized regression methods to address collinearity, we consulted extensively with our biostatistics department in preparation for our response to this comment. While there is moderate to strong positive correlation between total caloric intake and cholesterol intake (the two precision or control variables in the model, Rho=0.71), there was very weak correlation between categorized BCAA/1000 Kcal with either caloric intake (Rho= -0.08) or cholesterol intake (Rho= 0.17). When the exposure variable of interest is not collinear with control variables, the final coefficients or hazard ratios for the exposure variables are unlikely to be affected when these control variables are part of the model. Instead of formally testing this using Lasso regression, in the table below, we show the hazard ratios for the exposure variable with four models including or excluding total calories or cholesterol – demonstrating minimal difference among them. Our biostatistics consultants felt the standard Cox PH model can provide confidence intervals and p-values more easily (and automatically), whereas it is challenging to produce inference measures in LASSO. Exactly how to derive inferences in regularized regression in fact remains an open area of methodology research (Chatterjee et al. J American Statistical Association 2011 v106: p608-625; and <https://bookdown.org/tpinto_home/Regularisation/lasso-regression.html>, section 4.1).

Table. Adjusted risk (hazard ratio) of liver-related death or transplantation according to quartiles of BCAA intake derived from average daily energy adjusted BCAA intake (measured in grams of BCAA per 1000 kcal of daily energy intake). Results of four models to assess effect of collinear dietary variables (calories and cholesterol) on the estimates.

| Quartiles and range (g/1000 kcal of energy) of BCAA intake | ^a^aHR  (95% CI) | ^b^aHR  (95% CI) | ^c^aHR  (95% CI) | ^d^aHR  (95% CI) |
| --- | --- | --- | --- | --- |
| 1  (3.0-5.6) | 1.00 | 1.00 | 1.00 | 1.00 |
| 2  (5.6-6.4) | 0.92  (0.49-1.75) | 0.94  (0.50-1.77) | 0.92  (0.49-1.74) | 0.94  (0.50-1.77) |
| 3  (6.4-7.4) | 0.97  (0.50-1.90) | 1.01  (0.54-1.90) | 0.97  (0.51-1.85) | 1.02  (0.54-1.90) |
| 4  (7.4-34.8) | 1.03  (0.52-2.05) | 1.08  (0.57-2.04) | 1.03  (0.54-1.98) | 1.08  (0.57-2.04) |

^a^Adjusted for age, sex, race, BMI, diabetes, lifetime alcohol intake, smoking status, coffee intake, self-reported health status, cirrhosis status, duration of infection, peginterferon treatment group, daily average energy intake and daily average cholesterol intake.

^b^Adjusted for age, sex, race, BMI, diabetes, lifetime alcohol intake, smoking status, coffee intake, self-reported health status, cirrhosis status, duration of infection, peginterferon treatment group, daily average energy intake only without cholesterol.

^c^Adjusted for age, sex, race, BMI, diabetes, lifetime alcohol intake, smoking status, coffee intake, self-reported health status, cirrhosis status, duration of infection, peginterferon treatment group, daily average cholesterol intake without calories.

^d^Adjusted for age, sex, race, BMI, diabetes, lifetime alcohol intake, smoking status, coffee intake, self-reported health status, cirrhosis status, duration of infection, peginterferon treatment group only without calories or cholesterol.

Minor comments
1. The primary and secondary outcomes of this study included liver-related death, liver transplantation, presence of HCC and HE. Considering the enrolled patients were only with advanced fibrosis or compensated cirrhosis, the incidence of hepatic decompensation, including ascites formation, spontaneous bacterial peritonitis, variceal bleeding, and so on is suggested to investigate. Besides, the definition of liver-related death is needed to be clarified.

**Response**: The table below describes the rates of clinical decompensation events and we have included this table in the supplemental material. Each patient may experience multiple events. The HALT-C data did not have time-to-event data for each of these decompensations separately. But there was no statistically significant difference among the four BCAA intake categories for each of these events. HALT-C data did, however, report a composite decompensation variable which included time to the development of first decompensation including variceal bleeding, ascites, spontaneous peritonitis or encephalopathy. This data are included in our response to the Minor comment below and in the revised Results section of the manuscript.

In the Methods section, we have also provided clarification for the definition of liver-related death according to the latest HALT-C publication on mortality in the trail (Ref 24: Di Bisceglie et al. Hepatology 2011: v53, p1100-1108). Liver-related death is defined as death from end stage liver disease progression, liver cancer or Peg-interferon treatment induced decompensation as judged by a 7-person central review committee using the following likelihood scores: “unlikely” or <25% likelihood, “possible” or 25-49% likelihood, “probable” or 50-75% likelihood, and “highly likely” or >75% likelihood. When the likelihood is in the “probable” or “highly likely” category, the cause of death would be categorized as liver-related.

Table. Liver related decompensations according to quartiles of BCAA intake derived from average daily BCAA intake (measured in grams of BCAA per 1000 kcal of daily energy intake).

| Quartiles and range (g/1000 kcal of energy) of BCAA intake | Variceal bleeding  (N, %) | Ascites  (N, %) | Spontaneous peritonitis  (N, %) |
| --- | --- | --- | --- |
| 1  (3.0-5.6) | 5  (3.0%) | 18  (11%) | 1  (0.6%) |
| 2  (5.6-6.4) | 1  (0.6%) | 11  (6.7%) | 0 |
| 3  (6.4-7.4) | 4  (2.4%) | 16  (9.8%) | 2  (1.2%) |
| 4  (7.4-34.8) | 6  (3.7%) | 10  (6.1%) | 1  (0.6%) |

2. The crude hazard ratio of each factor that had been input into the regression model for the analyses of the risk of liver-related death, liver transplantation or liver decompensation should be provided in the table. The crude and adjusted risks of each factor to the presence of hepatic decompensation or HCC are also needed to be provided in the separate tables.

**Response**: In the following two tables, we included the crude and adjusted hazard ratios of variables from the original full model on: 1) liver related death or transplantation and 2) first hepatic decompensation event including: variceal bleeding, ascites, peritonitis or encephalopathy. As we noted in our response to minor comment 1 above, HALT-C did not have time-to-event data for each individual decompensations separately. But it did include time to the development of “first” decompensation including variceal bleeding, ascites, spontaneous peritonitis or encephalopathy as a composite endpoint. Both tables are included in the Supplemental Material.

Table 1. Crude and adjusted hazard ratios of liver-related death or transplantation according to quartiles of BCAA intake derived from average daily energy adjusted BCAA intake (measured in grams of BCAA per 1000 kcal of daily energy intake).

|  | Crude HR  (95% CI) | ^a^AHR  (95% CI) |
| --- | --- | --- |
| BCAA intake categories |  |  |
| 1 | 1 | 1 |
| 2 | 1.04 (0.60-1.81) | 0.92 (0.49-1.75) |
| 3 | 1.03 (0.59-1.80) | 0.97 (0.50-1.90) |
| 4 | 0.97 (0.55-1.70) | 1.03 (0.52-2.05) |
| Age | 1.01 (0.98-1.04) | 1.01 (0.97-1.04) |
| Sex |  |  |
| Male | 1 | 1 |
| Female | 0.85 (0.54-1.34) | 0.82 (0.46-1.47) |
| Race |  |  |
| White | 1 | 1 |
| Black | 1.30 (0.78-2.17) | 0.97 (0.51-1.85) |
| Hispanic | 1.01 (0.44-2.33) | 0.76 (0.29-1.97) |
| Other | 1.86 (0.67-5.10) | 1.97 (0.59-6.60) |
| Cirrhosis | 3.04 (2.00-4.63)^b^ | 2.96 (1.84-4.74)^b^ |
| Diabetes | 0.84 (0.49-1.44) | 0.91 (0.47-1.75) |
| Lifetime number of alcohol drinks | 1.00 (1.00-1.00) | 1.00 (0.94-1.04) |
| Body mass index | 0.99 (0.95-1.03) | 0.99 (0.94-1.04) |
| Smoking | 1.50 (1.01-2.22)**^b^** | 1.52 (0.94-2.48) |
| Self-rated health status |  |  |
| Excellent | 1 | 1 |
| Very good | 3.32 (0.44-24.7) | 3.80 (0.49-29.6) |
| Good | 4.11 (0.57-29.8) | 4.08 (0.53-31.1) |
| Fair | 3.89 (0.52-28.8) | 3.94 (0.50-31.1) |
| Poor | 5.48 (0.64-47.0) | 7.34 (0.78-69.3) |
| Peg-Interferon treatment | 1.01 (0.68-1.50) | 0.97 (0.62-1.51) |
| Duration of HCV infection | 1.01 (0.99-1.04) | 1.01 (0.98-1.04) |
| Coffee intake |  |  |
| Nondrinker | 1 | 1 |
| < 1 cup/day | 1.70 (0.90-3.21) | 1.73 (0.85-3.51) |
| 1-2 cups/day | 1.06 (0.56-2.00) | 1.01 (0.49-2.09) |
| ≥ 3 cups/day | 0.86 (0.34-2.15) | 0.78 (0.29-2.14) |
| Calorie intake | 1.00 (1.00-1.00) | 1.00 (1.00-1.00) |
| Cholesterol intake | 1.00 (1.00-1.00) | 1.00 (1.00-1.00) |

^a^Full model adjusted for age, sex, race, BMI, diabetes, lifetime alcohol intake, smoking status, coffee intake, self-reported health status, cirrhosis status, duration of infection, peginterferon treatment group, daily average energy intake and daily average cholesterol intake.

^b^ Indicates statistical significance at P<0.05

Table 2. Crude and adjusted hazard ratios of first liver related decompensations (first event of variceal bleeding, ascites, spontaneous peritonitis or encephalopathy) according to quartiles of BCAA intake derived from average daily BCAA intake (measured in grams of BCAA per 1000 kcal of daily energy intake).

|  | Crude HR  (95% CI) | ^a^AHR  (95% CI) |
| --- | --- | --- |
| BCAA intake categories |  |  |
| 1 | 1 | 1 |
| 2 | 0.70 (0.35-1.38) | 0.63 (0.30-1.36) |
| 3 | 1.10 (0.60-2.03) | 1.05 (0.51-2.16) |
| 4 | 0.97 (0.52-1.82) | 0.91 (0.42-1.95) |
| Age | 0.99 (0.96-1.03) | 0.99 (0.95-1.03) |
| Sex |  |  |
| Male | 1 | 1 |
| Female | 1.27 (0.78-2.05) | 1.07 (0.58-1.96) |
| Race |  |  |
| White | 1 | 1 |
| Black | 1.31 (0.72-2.38) | 1.18 (0.58-2.43) |
| Hispanic | 2.08 (1.02-4.22)^b^ | 1.04 (0.44-2.45) |
| Other | 0.60 (0.08-4.37) | 0.83 (0.11-6.25) |
| Cirrhosis | 3.26 (2.02-5.29)^b^ | 4.27 (2.44-7.48)^b^ |
| Diabetes | 0.95 (0.50-1.81) | 1.09 (0.52-2.28) |
| Lifetime number of alcohol drinks | 1.00 (1.00-1.00) | 1.00 (1.00-1.00) |
| Body mass index | 1.05 (1.01-1.09) | 1.03 (0.99-1.08) |
| Smoking | 0.88 (0.52-1.48) | 0.82 (0.44-1.51) |
| Self-rated health status | 1.57 (1.21-2.04)^b^ | 1.44 (1.06-1.96)^b^ |
| Peginterferon group | 1.31 (0.83-2.07) | 1.06 (0.64-1.77) |
| Duration of HCV infection | 0.99 (0.96-1.02) | 0.99 (0.96-1.02) |
| Coffee intake |  |  |
| Nondrinker | 1 | 1 |
| < 1 cup/day | 0.67 (0.34-1.28) | 0.60 (0.30-1.23) |
| 1-2 cups/day | 0.57 (0.31-1.05) | 0.54 (0.27-1.09) |
| ≥ 3 cups/day | 0.43 (0.16-1.17) | 0.42 (0.14-1.28) |
| Calorie intake | 1.00 (1.00-1.00) | 1.00 (1.00-1.00) |
| Cholesterol intake | 1.00 (1.00-1.00) | 1.00 (1.00-1.00) |

^a^Full model adjusted for age, sex, race, BMI, diabetes, lifetime alcohol intake, smoking status, coffee intake, self-reported health status, cirrhosis status, duration of infection, peginterferon treatment group, daily average energy intake and daily average cholesterol intake.

^b^ Indicates statistical significance at P<0.05

**Reviewer #2**: General comments
The study by Yu, et al. evaluated retrospectively the effect of dietary branch chain amino acids (BACC) on the liver- related outcomes including liver-related death, liver transplantation, hepatoma (HCC) development and hepatic encephalopathy (HE) in a HALT-C cohort of patients with advanced fibrosis or cirrhosis. In a cohort of 656 patients stratified into four groups by BCAA intake, there are no significant differences of the incidences of liver-related death, liver transplantation, HCC development and HE in a median follow-up of 5 years. Contrary to previous studies using BCAA as supplements of diet, this study estimated the BCAA contents of patients’ regular diet to investigate its impact on liver-related outcomes. Although interesting, there are some issues to be clarified.

Major comments
1. It is important to estimate of BCAA amount from the patients’ diet in this study. In the method section, the author might explain briefly how they calculated the BCAA amount from patients’ diet in the food questionnaires.

**Response**: The Block food frequency questionnaires (FFQ) estimate the amount of specific nutrients primarily based on the United States Department of Agriculture (USDA) Database for Standard Reference, Release 27 (USDA SR-27). These nutrient values were then combined with population and consumption-weighted intake data from NHANES (2007-2010) 24-hour dietary recalls, in order to determine the amount of nutrients found in the food or beverage in the questionnaire response. The average intake of a nutrient from a specific food item is the product of reported frequency of intake, reported portion size and the estimated nutrient amount from USDA and NHANES.

The above description was added to the Methods section.

2. Are the incidences of liver- related outcomes comparable to the similar patient cohorts outside HALT-C trial? This issue might be provided in the discussion section.

**Response**: The table below shows that the rates of hepatic decompensation, liver cancer and deaths from the current study were largely consistent with previous cohorts from western countries with similar length of follow-up. Because the focus of the current study is on dietary BCAA and outcomes, and none of these previous studies assessed diet, we did not include this information in the final manuscript.

| Study | Follow-up length | Rates of hepatic decompensation | Rates of liver cancer | Rates of mortality |
| --- | --- | --- | --- | --- |
| HALT-C  (United States) | 5 years | 13% | 8.5% | 12% |
| ^a^Fattovich et al. (Italy) | 5 years | 18% | 7% | 13% |
| ^b^Hu et al. (United States) | 4.5 years | 22% | 10% | 18% |

^a^Fattovich G, et al. Morbidity and mortality in compensated cirrhosis type C: a retrospective follow-up study of 384 patients. Gastroenterology 1997;112:463-472

^b^Hu K-Q, Tong MJ. The long-term outcomes of patients with compensated hepatitis C virus-related cirrhosis and history of parenteral exposure in the United States. Hepatology 1999;29:1311-1316

3. In addition to liver-related outcomes, the readers might also be interested in the all-cause and non-liver related death. The authors might provide these data.

Response: There were 32 non-liver related deaths during the study period. Combined with liver-related deaths, the incidence of all-cause mortality and liver transplantation was 41.9 per 100 person-years. This information was included in the Results section in the revised manuscript.

4. Direct-acting antivirals (DAA) will have a great impact on the liver-related outcomes. Are these patients undergoing DAA for hepatitis C virus treatment?

Response: Recent studies have confirmed the beneficial effect of DAA on liver related outcomes in HCV infected patients. The HALT-C trial took place before DAA became available, and no patients in the current study underwent DAA treatment.

5. Whether sarcopenia has an impact of the liver-related outcomes in this cohort might be analyzed in this study.

Response: Unfortunately the HALT-C data did not include anthropometric measurements of study patients. Because all study subjects had either advanced fibrosis or compensated cirrhosis at the time of enrollment, we suspect most patients were not sarcopenic. It would be interesting and important to assess age, sex and height adjusted muscle store to disease outcomes as the reviewer suggested in future studies.
